# Synthesis of Nanoscale TiO_2_ and Study of the Effect of Their Crystal Structure on Single Cell Response

**DOI:** 10.1100/2012/498345

**Published:** 2012-05-01

**Authors:** Z. R. Ismagilov, N. V. Shikina, N. A. Mazurkova, L. T. Tsikoza, F. V. Tuzikov, V. A. Ushakov, A. V. Ishchenko, N. A. Rudina, D. V. Korneev, E. I. Ryabchikova

**Affiliations:** ^1^Boreskov Institute of Catalysis, Siberian Branch of RAS, 5, Pr. Akad. Lavrentieva, Novosibirsk 630090, Russia; ^2^The Vector State Research Center of Virology and Biotechnology, Rospotrebnadzor, Korp. 12, Novosibirsk oblast, Koltsovo 630559, Russia; ^3^Institute of Chemical Biology and Fundamental Medicine, Siberian Branch of RAS, 8, Pr. Akad. Lavrentieva, Novosibirsk 630090, Russia

## Abstract

To study the effect of nanoscale titanium dioxide (TiO_2_) on cell responses, we synthesized four modifications of the TiO_2_ (amorphous, anatase, brookite, and rutile) capable of keeping their physicochemical characteristics in a cell culture medium. The modifications of nanoscale TiO_2_ were obtained by hydrolysis of TiCl_4_ and Ti(i-OC_3_H_7_)_4_ (TIP) upon variation of the synthesis conditions; their textural, morphological, structural, and dispersion characteristics were examined by a set of physicochemical methods: XRD, BET, SAXS, DLS, AFM, SEM, and HR-TEM. The effect of synthesis conditions (nature of precursor, pH, temperature, and addition of a complexing agent) on the structural-dispersion properties of TiO_2_ nanoparticles was studied. The hydrolysis methods providing the preparation of amorphous, anatase, brookite, and rutile modifications of TiO_2_ nanoparticles 3–5 nm in size were selected. Examination of different forms of TiO_2_ nanoparticles interaction with MDCK cells by transmission electron microscopy of ultrathin sections revealed different cell responses after treatment with different crystalline modifications and amorphous form of TiO_2_. The obtained results allowed us to conclude that direct contact of the nanoparticles with cell plasma membrane is the primary and critical step of their interaction and defines a subsequent response of the cell.

## 1. Introduction

Nanoscale titanium dioxide (TiO_2_) attracts considerable attention due to its unique physicochemical and catalytic properties, which found application in various fields including photocatalysis, electrochemistry, synthesis of catalysts and supports for environmental remediation, and production of pigments, cosmetics, and medicinal preparations [1–10]. Pigment-grade form of TiO_2_ (spheres larger than 200 nm) was considered safe for humans for a long time and widely used in pharmaceutical compositions and cosmetics [11, 12]. Usage of nanoscale TiO_2_ in these fields seems promising, so the possibility of nanoscale TiO_2_ application in therapy [13–15] and microbiology [16–18] was examined. The ability of 4-5 nm TiO_2_ nanocomposites including those with oligonucleotides to penetrate into cells [19–21] served as a background to expect that nanoscale TiO_2_ can provide a new class of advanced biologically active substances. However, there is no complete sureness in biological safety of nanoscale TiO_2_. Some studies showed that nanoscale TiO_2_ kept its inert properties in animals [22, 23] and humans [11, 24]. In contrast, other researchers found a clear toxic effect of nanoscale TiO_2_ both for cell cultures and animals [25–30]. Most publications relate toxic effects of TiO_2_ nanoparticles with their intrinsic capacity to generate reactive oxygen species (ROS), which could damage cell structures [31, 32]. Toxic effect of TiO_2_ nanoparticles depends on their shape, size, agglomeration state, surface chemistry, and concentration [31, 33, 34], and some papers reported different influence of rutile and anatase TiO_2_ on cell viability [25–28, 30]. Indeed, the size of nanoparticles allows them to approach the cell surface at distances of 1–100 nm, thus providing fundamentally new types of interaction of inorganic substances with the cell surface [35, 36]. The problem to catch and study these interactions makes special requirements for the stability of nanoscale TiO_2_ physicochemical properties. The requirements on the stability of physicochemical properties of nanoscale TiO_2_ sharply increase when they are used in biological studies. The need of physiological pH (7.2–7.4) and water-soluble preparations can cause great difficulties in the synthesis of nanoparticles. In addition, nanoparticles should be dissolved in physiological solutions for biological experiments, and this can induce agglomeration and changes of their morphology [37, 38]. It is evident that synthesis of nanoscale TiO_2_ must provide high level of their physicochemical stability sufficient for biological experiments.

Nanoscale TiO_2_ sols are synthesized mostly by two kinds of the sol-gel method differing in the chemical nature of titanium precursor: (1) with the use of alkoxides (most often, titanium isopropoxide or isobutoxide) and (2) with titanium tetrachloride, TiCl_4_ [39]. The goal of the present work was to obtain nanoscale TiO_2_ sols having stable physicochemical properties suitable for biological studies. To achieve this goal, we examined the effect of precursor nature and synthesis conditions on the textural, structural, and dispersion properties of titanium dioxide nanoparticles and their stability. The TiO_2_ nanoparticles were also characterized by a set of physicochemical methods (XRD, IR spectroscopy, BET, SAXS, DLS, SEM, TEM, AFM). We were able to choose the optimal conditions for preparation of different forms of nanoscale TiO_2_ with similar dispersion characteristics and stable morphology for the study of the influence of their modification on cell responses.

The most interesting questions are how discrete nanoparticles interact with cell plasma membrane and is there any difference in this interaction between different forms of TiO_2_? To answer these questions we used electron microscopy which allows us to see and study both nanoparticles and cellular structures in the nanometer scale. Several electron microscopic studies have been performed with commercial TiO_2_ (10–50 nm) nanoparticles and cells using a period of 24–48 h incubation [30, 40–42]. As shown in our previous studies, it is precisely the early steps of the nanoscale TiO_2_ interaction with a cell that can determine the cell response to nanoparticles [43]. Here, we report principal data of electron microscopic study on the interaction of 3–5 nm TiO_2_ of different modifications (amorphous, anatase, rutile, and brookite) with a single cell.

## 2. Materials and Methods

### 2.1. Materials

Chemicals and materials were obtained from commercial suppliers: TiCl_4_ (99.6%), Ti(i-OC_3_H_7_)_4_ (98%) (Alfa Aesar), 2-isopropanol, acetylacetone (99%) (Sigma-Aldrich), NaOH, HNO_3_ (Merck), cellulose membrane (ZelluTrans T1, Roth).

### 2.2. Preparation of TiO_2_ Nanoparticles

Samples of series 1 were obtained by simultaneous batching of TiCl_4_ in hydrochloric acid HCl (5 mol/L) and ammonia solutions (3.5 mol/L) or a NaOH solution (2.5 mol/L) into reaction vessel with deionized water under intense stirring at a specified temperature of 4, 20, or 70°C and constant pH kept at 3 ÷ 4, 6 ÷ 7, or 9 ÷ 10. The resulting colloidal solutions with a loose white sediment were stirred for 1 h, kept at room temperature for 5 h, washed with deionized water to remove Cl^−1^ and Na^+1^(NH_4_^+1^) ions, and stored at 4°C (samples 1.1–1.9 in Table 1).

Samples of series 2 were obtained by hydrolysis of TiCl_4_ in cold water at 3-4°C and pH < 1. One sample was dialyzed against deionized water at 3-4°C up to pH values 3.3–3.5. This gave a transparent sol, which was stored at 4°C. Another sample after hydrolysis was diluted by 1.6 M HCl and heated to 70°C under constant stirring for 1–5 h. After 1 h heating, the solution turned into a transparent sol, whereas the long heating resulted in a colloidal solution with sediment. The sediment was washed with deionized water at 3-4°C up to pH values 3.3–3.5 and stored at 4°C (samples 2.1–2.3 in Table 1). 

To synthesize series 3 samples, we used a mixture of titanium tetraisopropoxide (Ti(i-OC_3_H_7_)_4_, TIP) and isopropyl alcohol with the volume ratio of components 6 : 1; the mixture was added dropwise under intense stirring to an aqueous solution of nitric acid taking [H^+^]/[Ti] = 0.2 and [H_2_O]/[Ti] = 200 [15]. The sol was stirred for 7 h at a specified temperature 20, 50, 70, or 95°C and then cooled to room temperature. One sample was obtained by introduction of acetylacetone ([ACAC]/[Ti] = 2) [44] to the initial mixture of titanium tetraisopropoxide and isopropyl alcohol, other conditions of nitric acid hydrolysis being equal. The sol was stirred for 7 h at 60°C and then cooled to room temperature. The sols of series 3 were dialyzed against deionized water at 3-4°C up to pH values 3.3–3.5 and stored at 4°C (samples 3.1–3.5 in Table 1). 

For XRD and BET studies, samples were dried at room temperature and under an IR lamp at 70°C. The samples for SAXS, DLS, AFM, SEM, TEM, and HR-TEM studies were used without drying. For experiments with MDCK cell culture, the samples were neutralized and cleaned by dialysis. 

### 2.3. X-Ray Powder Analysis

X-ray diffraction (XRD) patterns were recorded over a 2*θ* = 20–85° range using an HZG-4C diffractometer (Freiberger Prazisionsmechanik, Germany) with CoK*_*α*_* (*λ* = 1.59021 Å) irradiation. The crystal sizes of TiO_2_ were estimated in accordance with Sherrer equation [45] applied to the (101) reflection at 2**θ**= 29.46° (anatase), the (121) reflection at 2**θ**= 35.95° (brookite), and the (110) reflection at 2**θ**= 31.0° (rutile). 

### 2.4. Nitrogen Adsorption

To analyze the textural characteristics of TiO_2_, low-temperature (77 K) nitrogen adsorption-desorption isotherms were recorded using a Micromeritics ASAP 2400 (Micromeritics, USA). The specific surface area S_BET_ was calculated according to standard BET method. The incremental pore and size distributions were calculated by the Barrett-Joyner-Halenda method. 

### 2.5. Scanning Electron Microscopy (SEM)

The study of TiO_2_ morphology was performed using a JSM 6460LV (JEOL, Japan) scanning electron microscope with accelerating voltage 25 kV. Samples were fixed on a copper holder with conductive glue or scotch tape. Preliminarily, a 5–10 nm thick conductive layer of gold was sprayed on the samples surface in a special vacuum unit to eliminate charging effects. 

### 2.6. Small-Angle X-Ray Scattering (SAXS)

SAXS patterns from the samples of colloidal solutions and sols were measured on the small-angle X-ray diffractometers (Siemens, Germany and Hecus, Austria), which provide sample thermostabilization within 0 to 70°C and the accuracy of ±0.1°C. X-ray tubes with copper anode were used as a source of irradiation (*λ*_Cu*K*_*α*__ = 1.54 Å). 

For analysis, samples were placed in a special cuvette made of 1 mm thick quartz capillary with 0.01 mm walls. As a background scattering we measured scattering from cuvettes filled with the liquid phase whose composition was close to that of mother liquor of the sol under study. SAXS measurements were made at 24°C over the angle range 2*θ* = 0.17 ÷ 2.34° (adjusted for absorption of X-rays, collimation of X-ray beam and background scattering). 

After instrumental correction, the obtained experimental SAXS data were used to calculate the functions of TiO_2_ nanoparticle size distribution (inhomogeneities of electron density) (*D*_v_(*R*)) in the form of histograms, prevailing radius of the particles (*R*), and specific surface area (*S*/*V*). To determine *S*_SAXS_ referred to TiO_2_ weight, the value of *S/V* was corrected with regard to the true density of different titanium dioxide modifications, which is equal to 3.95 g/cm^3^ for anatase, 4.10 g/cm^3^ for brookite, 4.23 g/cm^3^ for rutile, and 3.39 g/cm^3^ for amorphous TiO_2_. 

### 2.7. Dynamic Laser Scattering (DLS)

Hydrodynamic diameter of TiO_2_ particles was measured with a NICOMP 380ZLS instrument (Particle Sizing Systems, USA). The instrument calculates diffusivity and size of the particles by equation (*D = kT/6*πη*R*, where *D* is hydrodynamic diameter of the particles; *k* is diffusivity of the particles; *T* is temperature; **η** is viscosity of solution; *R* is gas constant). Measurements were made in a 1 × 1 × 5 cm cuvette at 23°C, which corresponds to viscosity index for water 0.933 cP and refractive index 1.333; signal accumulation time was 10 min. 

### 2.8. Atomic Force Microscopy (AFM)

Atomic force microscopy images were carried out on a SolverP47Bio atomic force microscope (Zelenograd, Russia) in a semicontact mode. Samples were diluted in deionized water to a desired concentration. Then, a 20 *μ*L drop was placed on a fresh mica sheet with the surface area of ca. 25–30 mm^2^. Adsorption was carried out for 1 h at room temperature, and then residual liquid was removed with filter paper.

### 2.9. High-Resolution Transmission Electron Microscopy (HR-TEM)

HR-TEM images were taken on a JEM 2010 electron microscope (JEOL, Japan) with lattice resolution 0.14 nm at accelerating voltage 200 kV. Samples were deposited by an ultrasonic disperser onto standard carbon-coated copper grids, which were fixed in a holder and introduced in the chamber of electron microscope.

### 2.10. Transmission Electron Microscopy of MDCK Cell Culture Treated with Nanoscale TiO_2_ (TEM)

MDCK cell culture originated from Madin Darby canine kidneys and are epitheliod cells growing in monolayer. MDCK cell culture for electron microscopy was seeded on 6-well plates at a density of 1.0–1.5 × 105 cells/mL in 3 mL culture medium to each well and was propagated to full monolayer confluence in SFM4MegaVir (Invitrogen, Paisley, UK) cell culture medium supplemented with 2% Gibco fetal bovine serum (USA), at 37°C in the presence of 5% CO_2_, 100 U/mL of penicillin, and 100 *μ*g/mL streptomycin. The nanoparticles were diluted to 100 and 300 *μ*g/mL with the same cell culture medium. The cells were treated with amorphous, anatase, rutile, and brookite TiO_2_ nanoparticles in both concentrations after washing with fresh cell culture medium. Then the cells were incubated during 1, 3, and 5 h at 37°C in the dark, washed with fresh cell culture medium, collected in plastic tubes, and centrifuged at 3000 rpm. The pellets were fixed by 4% paraformaldehyde, routinely processed for electron microscopy, and embedded in an Epon-Araldite resin mixture. Ultrathin sections were routinely stained with uranyl acetate and lead citrate and examined on a JEM 1400 (JEOL, Japan) transmission electron microscope at 80 kV. Digital images were collected by Veleta 3 CCD camera (SIS, Germany). 

## 3. Results and Discussion 

Synthesis conditions of freshly prepared TiO_2_ sols and their textural and structural-dispersion properties such as pore volume *V*_pore_, specific surface area *S*_BET_, phase composition, and particle size are presented in Table 1. 

### 3.1. XRD Analysis

All the samples were tested by XRD method. Radiographic examination showed that phase state and particle size of TiO_2_ depend on conditions of hydrolysis of the both type precursors. At hydrolysis of TiCl_4_ (series 1), the dependence of TiO_2_ structural modification on pH of reaction medium is highly pronounced. Neutral or alkaline medium yields X-ray amorphous titanium dioxide. The pH value equal to 3-4 leads to preferential formation of the anatase phase with the particle size 4–6 nm; traces of brookite with the particle size less than 3 nm are observed. The amount of the brookite phase increases with a decrease in the synthesis temperature. As pH decreases below 1 (series 2), this results in the formation of brookite, which turns into rutile upon long-term heating at 70°C. The hydrolysis of Ti(i-OC_3_H_7_)_4_ under varying temperature (series 3) provides formation of the anatase phase, whose particle size has a weak tendency to increase with elevating temperature of the synthesis. TIP modification with acetylacetone (sample 3.5) produces no changes in the phase composition of hydrolysis products and facilitates the formation of the most dispersed particles of the anatase. 

**Figure 1:**
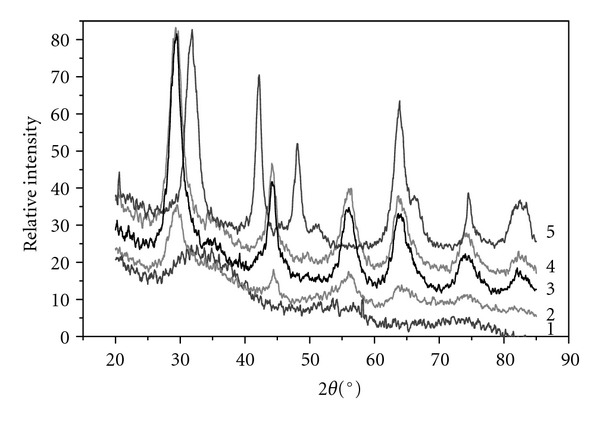
Diffraction patterns of titanium dioxide samples of different structure: (1) X-ray amorphous, (2) brookite, (3) anatase (from TiCl_4_), (4) anatase (from TIP), and (5) rutile.

Diffraction patterns of some samples with different structure are presented in Figure 1. The pattern of X-ray amorphous titanium dioxide (sample 1.5) shows a broad peak in the angle region 25–40°, which is typical of this phase. Brookite has a peak in the region of 29.53–35.95° (sample 2.1); however, the presence of anatase cannot be ruled out in this sample, its most strong line being superimposed with the brookite line in the region of 29.46°. Main distinction of the brookite diffraction pattern from that of anatase is asymmetry of the peak in the region of 29–31° and appearance of line (121) at 36°. Other lines of anatase show peaks in the angle regions of 44.22, 45.14, 56.47, and 63.54°. These regions correspond to peaks on the curves of samples 1.7 and 3.3; diffraction patterns of the samples obtained from different precursors, TiCl_4_ and Ti(i-OPr)_4_, are identical. Sample 2.3 is characterized by peaks in the angle regions of 31.56, 42.11, 48.04, and 63.80°, which correspond to rutile according to JCPDS radiographic database. 

Our data showed that nanoscale TiO_2_ sols of different structure can be obtained with equal ease from titanium tetraisopropoxide or titanium tetrachloride used as a Ti precursor. pH of the medium and temperature were found to affect the formation of TiO_2_ structure. Low pH (3-4) results in the formation of anatase phase, whereas neutral and alkaline values of pH lead to X-ray amorphous TiO_2_. Hydrolysis of TiCl_4_ in cold water at pH <1 yields highly disperse brookite sols; long-term heating of brookite transforms it into rutile.

### 3.2. N_2_ Adsorption

**Figure 2:**
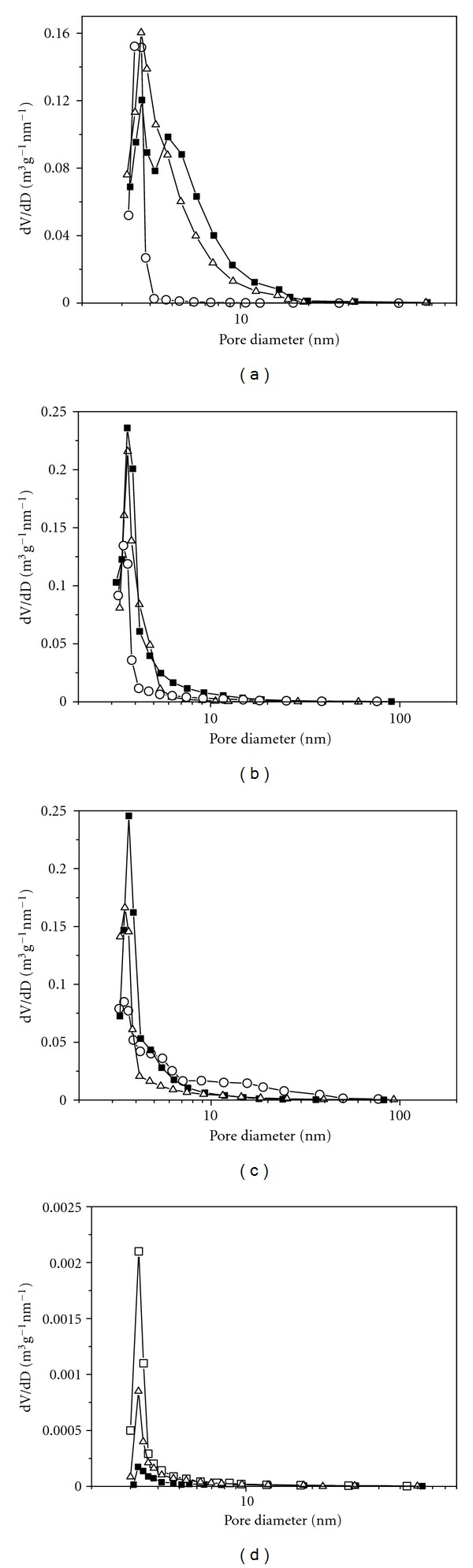
Differential curves of the pore size distribution in series 1 samples obtained by hydrolysis of TiCl_4_ under the following conditions: (a) samples 1.1–1.3, *T* = 4°C; (b) samples 1.4–1.6, *T* = 20°C; (c) samples 1.7–1.9, *T* = 70°C (pH: ○—3 ÷ 4, ■—6 ÷ 7, ∆—9 ÷ 10); and (d) series 3 samples obtained by hydrolysis of TIP (*T*_synthesis_: ■—50°C, ∆—70°C, □—95°C). Pore size distribution was determined by BET.

Differential curves of the pore size distribution in the dried samples of series 1 (Figures 2(a)–2(c)) indicate a fine-pore structure with the prevailing pore size of 3-4 nm. However, some of the samples obtained in neutral and alkaline medium (*T* = 4°C, Figure 2(a)—■, ∆) or in acid medium (*T* = 70°C, Figure 2(c)—○) have a broad set of pores with the size ranging from 3 to 30 nm. Therewith, in all temperature groups of the samples, specific surface area *S*_BET_ increases with raising the synthesis temperature and pH (Table 1), which agrees with the classic concept of fresh sediment texture formation. At the synthesis temperatures of 4 and 20°C, pore volume *V*_pore_ has a maximum at pH = 6-7, whereas at 70°C it decreases monotonically with increasing pH (Table 1). An increase in the pore volume and formation of heterogeneous pore structure are related with the formation of bigger particles at these conditions of synthesis, which is confirmed by the SAXS data (Table 1). Samples obtained by drying the sols of series 3, also irrespective of the synthesis temperature, have even a more distinct homogeneous pore structure with the pore diameter 1-2 nm (Figure 2(d)) and a small pore volume, which points to a compact packing of the particles with close sizes. Pore size distribution for sample 3.1 is not shown because of too low *dV/dD* values. According to BET data, samples of series 3 are characterized by a proportional increase of specific surface area and pore volume as the synthesis temperature rises from 20 to 95°C and particle size grows (Table 1). 

### 3.3. SEM

**Figure 3:**
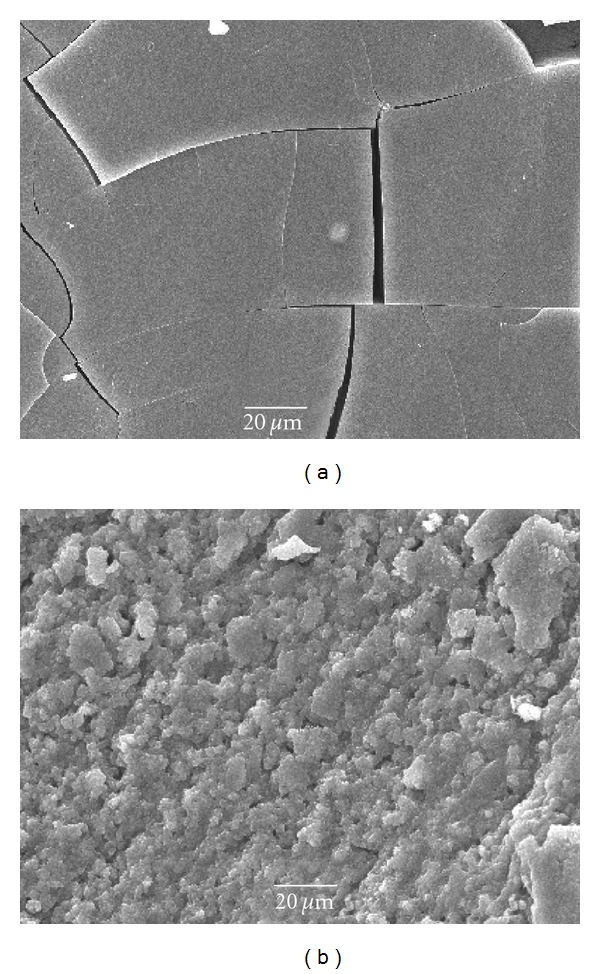
SEM images. Surface morphology of the dried samples of TiO_2_ sols obtained by hydrolysis of TIP at 20°C (a) and 95°C (b).

The BET data indicating that porosity of xerosols increases with elevation of the TIP hydrolysis temperature are supported by the SEM study of morphological structure of TiO_2_ films. The SEM images of dried sols, which were obtained at different temperatures of Ti(i-OC_3_H_7_)_4_ hydrolysis (samples 3.1 and 3.4), having the crystallite size 3.0 and 5.4 nm, respectively, are shown in Figure 3. The sample with small particle size (Figure 3(a)) has a vitreous morphology with practically nonporous structure (*S*_BET_ for this sample is very low, 2 m^2^/g, Table 1), whereas another sample shows a clearly defined porous structure (Figure 3(b)). 

### 3.4. SAXS

A comparison of specific surface areas determined by BET and SAXS methods shows that in some cases both methods give close values for samples of series 1 (1.3–420 and 400 m^2^/g; 1.4–350 and 330 m^2^/g) and sample 2.3 (175 and 200 m^2^/g); however, for highly dispersed sols the values of *S*_SAXS_ strongly exceed those of *S*_BET_ (samples 2.1, 3.3, 3.5, Table 1). These distinctions may be caused by ultramicropores in xerosols, which are inaccessible to nitrogen molecules at 77 K due to activated diffusion or their complete blocking (the presence of perfectly isolated pores). 

**Figure 4:**
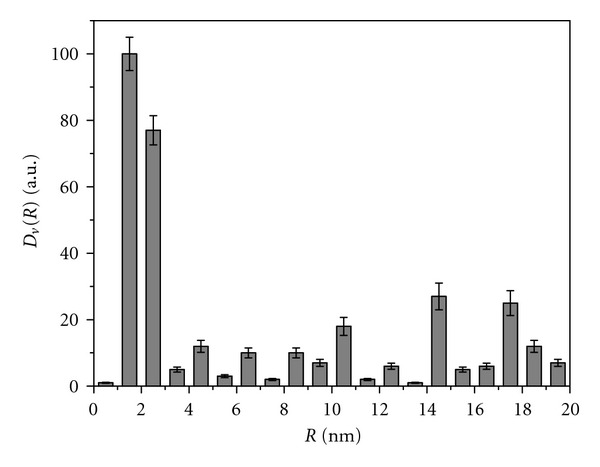
SAXS histogram of the particle size distribution in amorphous sample obtained by hydrolysis of TiCl_4_ in the presence of ammonia.

**Figure 5:**
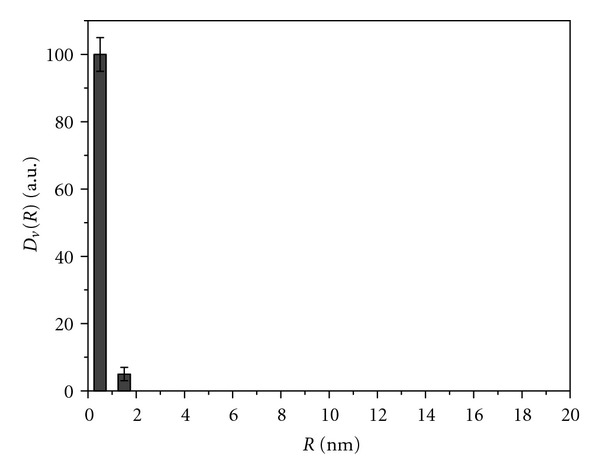
SAXS histogram of the particle size distribution in brookite sample obtained by hydrolysis of TiCl_4_ in cold water.

**Figure 6:**
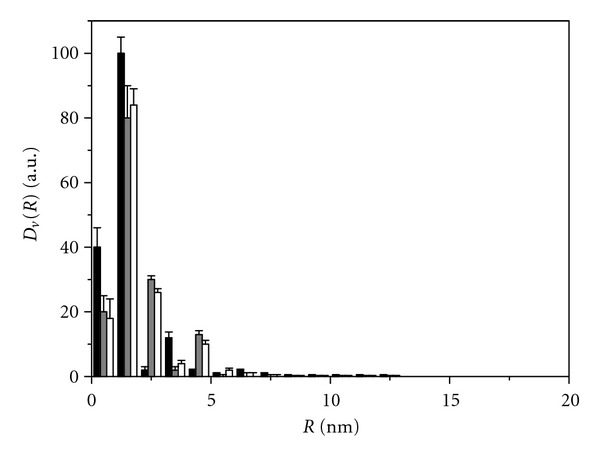
SAXS histogram of the particle size distribution in anatase samples obtained by hydrolysis of TIP in the presence of acetylacetone: black bars-initial sol after synthesis; grey bars-secondary sol obtained from dry initial sol after repulping in water; white bars-sol obtained from the secondary sol after centrifuging at 3000 rpm.

Examination of the particle size in TiO_2_ samples of all series revealed that, according to SAXS patterns (not shown), the analyzed TiO_2_ nanoparticles in sols are represented to a first approximation by equiaxed (with the axial ratio lower than 3 : 1) homogeneous spheres [46]. Histograms of the particle size distribution for series 1 samples, which are colloids with a sediment, are close to each other (Figure 4) and indicate the presence of TiO_2_ nanoparticles with the size of 4 to 40 nm and prevailing average diameter *D* = 5-6 nm (Table 1). Hydrolysis of TiCl_4_ in water at low pH and temperature 3-4°C (sample 2.1) results in the formation of a sol with virtually monodisperse particle size distribution (Figure 5). Histograms of all samples of series 3 indicate the presence of particles with size 2–20 nm and prevailing *D* = 3–5 nm. For sample 3.5 obtained by hydrolysis of Ti(i-OC_3_H_7_)_4_ in the presence of acetylacetone, even a more narrow distribution is observed (Figure 6): particles with the size above 10 nm are virtually absent, and prevailing diameter of the particles is equal to 4 nm. After drying and repulping of this sample, dispersion characteristics of the secondary and initial sols are almost identical. No significant changes of the characteristics were observed upon centrifugation of the secondary sol and particles sedimentation. 

Hydrolysis of TiCl_4_ in cold water and hydrolysis of TIP in acid medium yield highly disperse sols (brookite and anatase forms). However, the need of neutralization of TiO_2_ sols to reach the physiological pH (7.2–7.4) not far from its isoelectric point can entail agglomeration of the particles. In experiments with neutralized samples, the SAXS data indicated an increase in the mean diameter of brookite from 2.2 to 4 nm and marginal changes in the size of series 1 and 3 samples (not shown). 

It should be noted that due to high resolving power of SAXS method, the obtained data may reflect the size distribution of primary TiO_2_ nanoparticles separated by a boundary in the agglomerates. Thus, the question concerning the extent of particles agglomeration in the sols under study and the size of agglomerates still remains problematic.

### 3.5. DLS

The method of dynamic laser scattering, assessing the modulation frequency of scattered light from moving particles, can determine the hydrodynamic diameter of both the primary particles separated by a disperse medium and the aggregated entities. 

Table 2 shows the mean hydrodynamic diameter of different forms of TiO_2_ particles before and after their neutralization and cleaning by dialysis. Besides, to conclude more reliably on the presence or absence of TiO_2_ particles aggregation in the initial sols, all samples were tested after their ultrasonic treatment (US) at 22 kHz for 10 min. The hydrolysis of TiCl_4_ in the presence of alkalies (sample 1.5) yields large TiO_2_ agglomerates of the size exceeding 10 *μ*m (Table 2). The ultrasonic treatment decomposes these agglomerates into particles with the size of ca. 200 nm, which in their turn may be the agglomerates of 5-6 nm nanoparticles detected in these samples by SAXS (Table 1). DLS data for brookite well correlate with the XRD and SAXS measurements. This sol consists of small agglomerates with the prevailing size below 20 nm, which separate into 2.2 nm particles under the ultrasonic treatment. Rutile samples showed clear differences between DSL, SAXS, and XRD data: they were composed only from large particles regardless of the treatment conditions. TiO_2_ nanoparticles obtained by hydrolysis of titanium tetraisopropoxide have sizes typical of primary particles (3 nm, sample 3.5) or small agglomerates (7–10 nm, samples 3.2, 3.3). Ultrasonic treatment of these samples leads to a minor coarsening of the particles, probably due to an increase of their collision frequency in sols. 

A significant increase of hydrodynamic diameters for acid sols of highly disperse brookite and anatase was observed after their neutralization, whereas the large amorphous and rutile particles did not change. Anatase samples 3.3 and 3.5 formed similar agglomerations under neutral conditions (Table 2); however, sample 3.3 seems more attractive for subsequent operations because sample 3.5 contains acetylacetonate groups, which can affect the pattern of nanoparticles interaction with a cell. 

### 3.6. AFM

**Figure 7:**
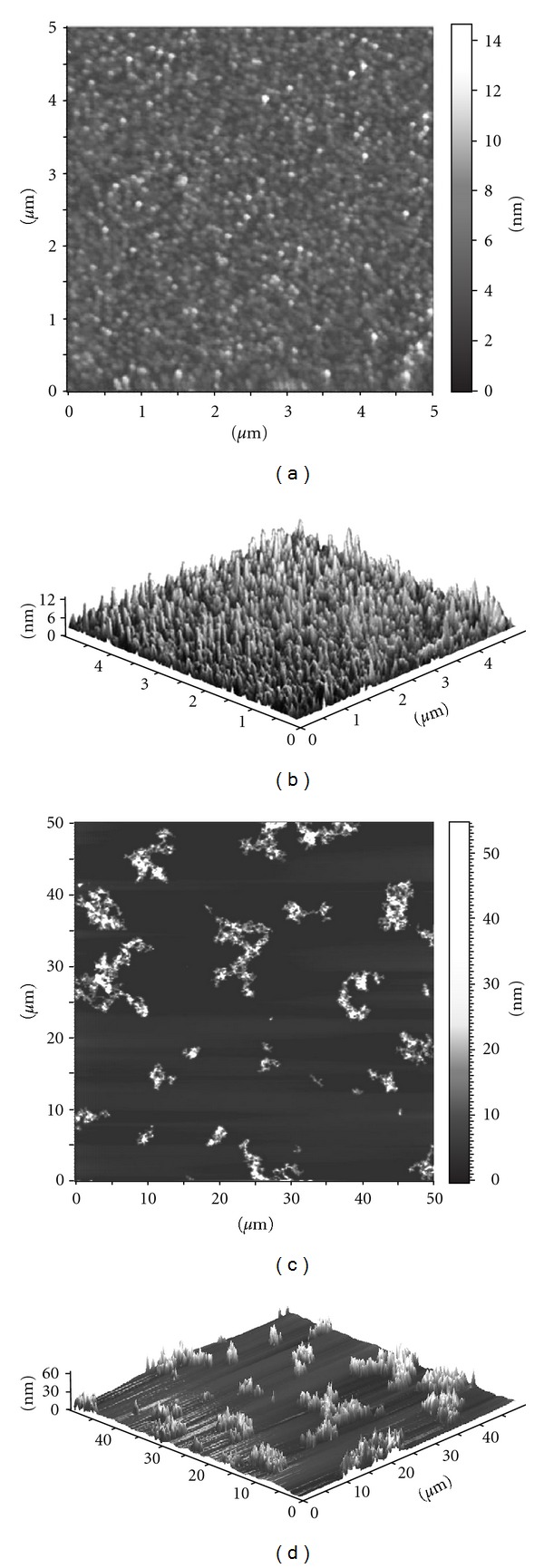
AFM images of anatase sample 3.3: 2D (a) and 3D (b) view of the freshly prepared acid sol; 2D (c) and 3D (d) view of the neutralized sol.

The results of AFM, which provides visualization of nanoparticles (Figure 7), fully confirmed the data obtained by other methods. AFM images of sample 3.3 (used as example) clearly showed that TiO_2_ acid sol (Figures 7(a) and 7(b)) consists of isolated particles with size 5–8 nm, while the neutralized sample consists of small chain-like agglomerates 20–60 nm in size (Figures 7(c) and 7(d)). 

### 3.7. TEM

**Figure 8:**
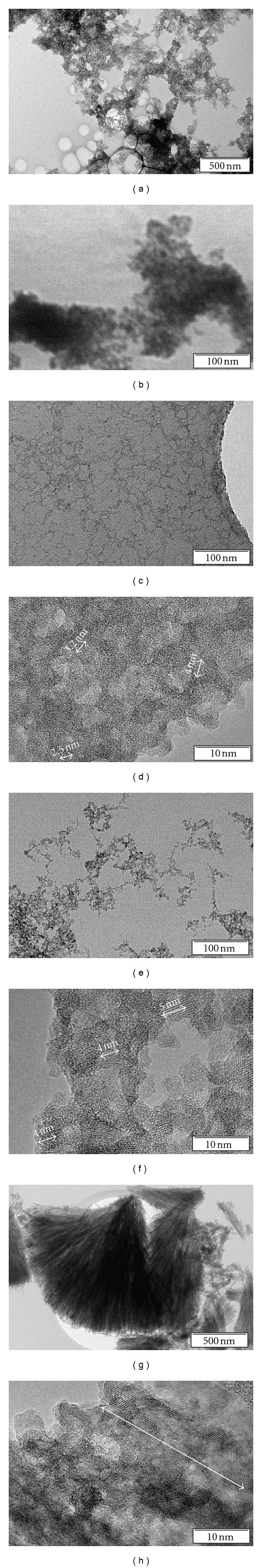
HR-TEM micrograph of nanoscale titanium dioxide samples of different structure: amorphous (a, b), brookite (c, d), anatase (e, f), and rutile (g, h). Arrows indicate planes of the aggregate formed by spliced particles. The needle crystals consist of coherently spliced crystallites having the size of 5-6 nm along plane (100) and 4-5 nm along plane (110).

**Figure 9:**
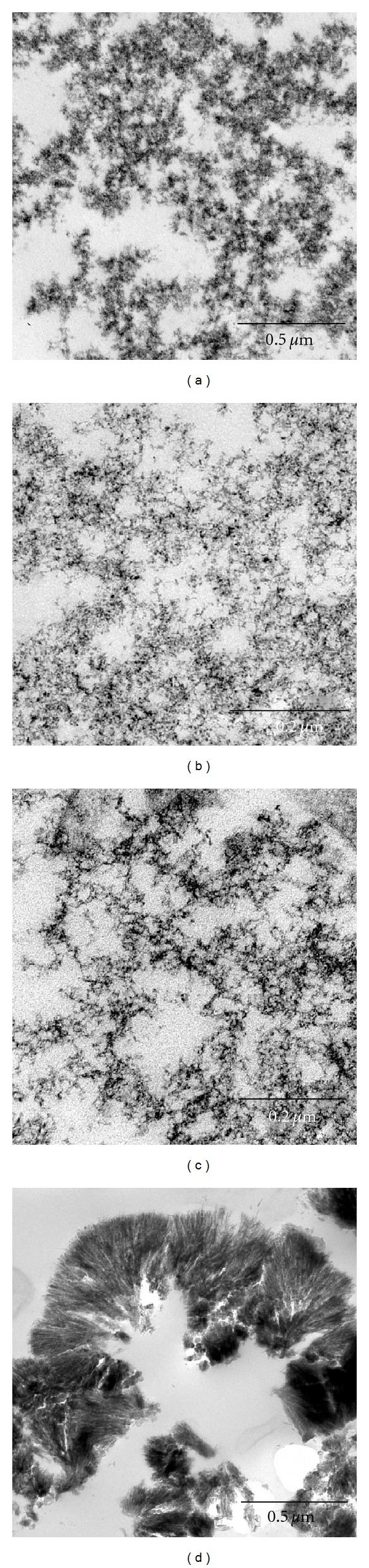
TEM images of TiO_2_ nanoparticles of different crystalline modification in ultrathin sections: amorphous (a), anatase (b), brookite (c), and rutile (d). Ultrathin sections.

Morphology and stability of different TiO_2_ forms in a cell culture medium amorphous (1.5), brookite (2.1), rutile (2.3), and anatase (3.3), were studied by transmission electron microscopy using routine dispersion (Figure 8) and ultrathin sections (Figure 9). The first allowed us to see particles of amorphous sample, which earlier were found to be large agglomerates (Figure 8(a)) consisting of the 5–10 nm particles (Figure 8(b)). The samples of brookite (Figure 8(c)) and anatase (Figure 8(e)) were represented by almost rounded nonagglomerated or weakly agglomerated particles with a narrow size distribution. Discrete small particles of brookite 3-4 nm in size and particles of anatase having a 4-5 nm size are presented in Figures 8(d) and 8(f). The rutile particles clearly differed in morphology from others: there were dendrites forming a “fan” of needle crystals 500–1500 nm in length (Figure 8(g)). Planes (110) are marked with an arrow in Figure 8(h). The needle crystals consist of coherently spliced crystallites having the size of 5-6 nm along plane (100) and 4-5 nm along plane (110). 

Thus, three crystal modifications of TiO_2_ (anatase, brookite, and rutile) have similar crystallite sizes (3–5 nm) equal to the particle size of amorphous sample. Although rutile is represented by strongly aggregated particles, diameter of the needle crystals is nanoscale and comparable with the size of other TiO_2_ forms. 

**Figure 10:**
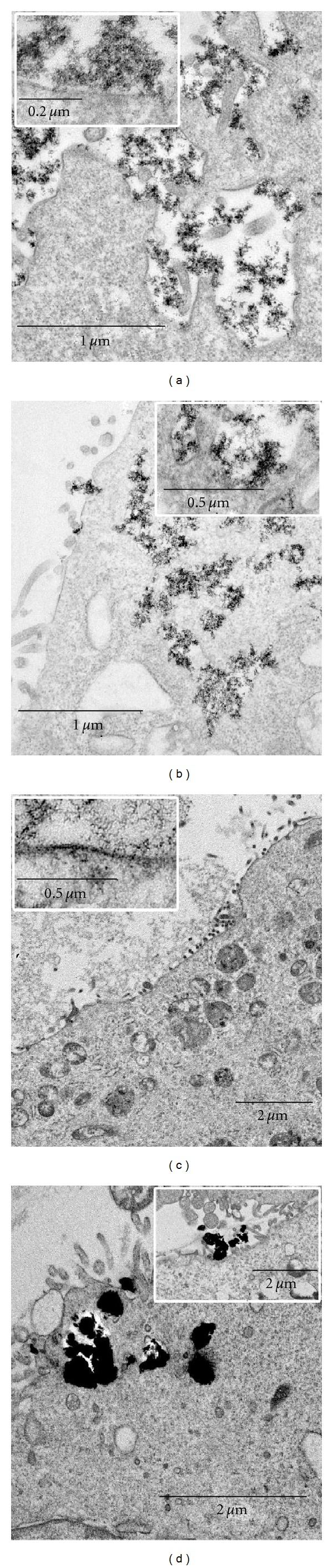
Interaction of MDCK cells with TiO_2_ nanoparticles: amorphous (a), anatase (b), brookite (c), and rutile (d). Inserts show direct contact of the nanoparticles with cell plasma membrane. Ultrathin sections. Transmission electron microscopy.

It was interesting to compare TEM images of nanoscale TiO_2_ dispersed on grids and the same particles in ultrathin sections (thickness of a section is 50–60 nm). Nanoscale TiO_2_ in ultrathin sections are more distinct, so it was possible to clearly discern single nanoparticles. Thus, amorphous nanoparticles had spherical shape and their size was 4-5 nm; rare particles had the size of 5–10 nm. Particles formed the band-like agglomerations with different electron density depending on the mass of particles in a section (Figures 9(a) and 10(a)). Anatase and brookite nanoparticles had distinct needle-like shapes, each needle lying separately and forming the branched structures that looked as a delicate lace (Figures 9(b), 10(b), 9(c), and 10(c)). Thus, the method of ultrathin sections gave an opportunity to easily recognize the morphology of anatase and brookite single particles and their spatial organization in comparison with dispersion on a grid, which is widely used in nanoparticle studies. Rutile morphology in ultrathin section was identical to those revealed by dispersion method (Figures 9(d) and 10(d)). The dendrites were 4-5 nm in diameter and formed aggregates resembling the palm-leaved or fan-like structures with sizes up to 30–50 nm. The appearance of rutile aggregates depended on the plane of ultrathin section, such aggregates could look formless. 

The morphology and sizes of all the examined forms of nanoscale TiO_2_ in ultrathin sections corresponded to those determined by other methods in this work. Our study demonstrated that treatment of the samples for ultrathin sectioning preserves the TiO_2_ nanoparticle morphology and the 1 h incubation with living cells has no effect on nanoparticles. It can be concluded that TiO_2_ samples prepared in this work are stable enough and the ultrathin sections method can be used successfully for examination of nanoparticle size and morphology. Moreover, it could be particularly useful for studies of very small nanoparticles (less that 5 nm). 

### 3.8. Electron Microscopic Study of Nanoscale TiO_2_ Interaction with MDCK Cells

Amorphous TiO_2_ nanoparticles in ultrathin sections covered large areas of MDCK cell surface and directly contacted with the cell surface, filled all folds and cavities formed by the cell plasma membrane, and thereby deeply penetrated into the cell after 1 h incubation (Figure 10(a)). More details of the interaction of amorphous and other modifications of TiO_2_ nanoparticles with the cell plasma membrane were described elsewhere [43]. 

Similar to amorphous form, the anatase TiO_2_ nanoparticles also penetrated into MDCK cells through folds and invaginations. The bulk of anatase directly contacted with the cell surface via single “needles” (Figure 10(b)). Some anatase nanoparticles entered the cells via the clathrin-mediated (receptor-mediated) endocytosis. The involvement of clathrin-mediated endocytosis in anatase internalization was shown recently by other authors [35, 47, 48]. Exploitation of the endocytosis pathway resulted in anatase accumulation in endosomes and phagosomes after 1 h incubation. 

Brookite TiO_2_ nanoparticles directly contacted with the plasma membrane via single needles in the same manner as anatase; however, brookite nanoparticles remained on the MDCK cell surface after 1 h incubation (Figure 10(c)). In contrast to amorphous and anatase TiO_2_, brookite-treated cells were rounded and did not have invaginations and folds. No brookite nanoparticles were found in endosomes and phagosomes. Many brookite-treated MDCK cells showed pathological changes, which progressed to necrosis. All damaged cells had close contact with brookite nanoparticles, but no nanoparticles were detected inside the cells. There are no publications in relevant literature describing the interaction of brookite TiO_2_ nanoparticles with cells or animals. Our study found that the nanoscale brookite possesses properties clearly differing from those of anatase and amorphous TiO_2_, despite identical sizes and similar shapes. 

Examination of MDCK cells treated with rutile nanoparticles for 1 h revealed aggregations located on the cell surface and in large and small vacuoles (Figure 10(d)). These vacuoles could form by buckling under the weight of rutile or perhaps by active cell uptake. We did not observe any contact of individual rutile particles with the cell surface; this TiO_2_ modification interacted with the cells like macroparticles. Rutile caused damage of some MDCK cells; however, their number was incomparably less than in brookite-treated samples. 

Thus, the identical size TiO_2_ nanoparticles, namely, amorphous, anatase, brookite, and rutile, showed different patterns of interaction with a cell. Incubation of MDCK cells with these nanoparticles for 3 and 5 h did not change the patterns; meanwhile, amorphous and anatase nanoparticles accumulated inside cells. Most of brookite-treated cells showed no signs of nanoparticles internalization after 3 h incubation, whereas few cells had small surface folds and invaginations containing the nanoparticles. Nanoparticles lying inside the invaginations were located closer to each other as compared to those lying outside the cells; nevertheless, there was no agglutination of the needles. MDCK cells penetrated with folds and invaginations containing the nanoparticles increased in number after 5 h incubation, whereas about 50% of the cells maintained their structure and did not internalize the nanoparticles. 

Incubation of MDCK cells with all studied modifications of TiO_2_ nanoparticles for 3 and 5 h resulted in a definite increase of the number of damaged cells. Toxic effects of these nanoscale TiO_2_ modifications on cells and their discussion were reported earlier [43]. 

Our study clearly demonstrated that transmission electron microscopy of ultrathin sections is a good tool to examine the interaction of individual nanoparticles with cells and evaluate their effects. We observed the direct contact of single TiO_2_ nanoparticles with the cell surface and the effect of nanoparticle modification on the nature of such contact. This means that nanoscale nonorganic substances are able to affect directly the cell functions probably due to their extremely close nanoscale contact with the cells. Distinct differences in cell response to different TiO_2_ nanoparticles could be attributed to two main factors. First, direct contact of amorphous, anatase, and brookite individual nanoparticles with the cell plasma membrane may alter its function by a simple mechanical binding to membrane macromolecules. Thus, contact with brookite could decrease the liquidity of the cell plasma membranes; they become “hardened” and unable to form invaginations. Amorphous and anatase TiO_2_ nanoparticles are likely to increase the liquidity of cell plasma membrane, allowing it to readily form deep invaginations providing the penetration of nanoparticles into the cell. 

Another mechanism of cell plasma membrane alteration could be related to chemical interaction between its macromolecules and TiO_2_ nanoparticles, which are able to generate free radicals, including oxygenated free radicals HO_2_^•^, O_2_^•^, HO^•^, and carbon-centered radicals, causing cleavage of C–H bonds in organic molecules. These reactions can occur even in the dark and serve as the first step of oxidative damage of biological molecules [49, 50]. The possibility of direct chemical interaction of TiO_2_ with the cell membrane molecules also cannot be excluded. 

Different modifications of TiO_2_ can induce different chemical reactions, thus leading to different alterations in macromolecular structure of the cell membrane. We believe that direct contact of TiO_2_ nanoparticles with the cell plasma membrane is the primary and basic step of cell damage, operating for all modifications of nanoparticles whose sizes allow direct contact with the membrane. Pathological changes found in MDCK cells treated with TiO_2_ nanoparticles represent an impairment of the water-ion balance, which could be the first consequence of plasma membrane damage by TiO_2_ nanoparticles and the start of alterations in other cellular pathways. The features of the interaction of TiO_2_ nanoparticles having different crystalline modifications with the cells identified in our work indicate the need of careful selection of the type of nanoparticles for construction of the means to influence a living cell, in particular for the delivery of biologically active substances. 

Thus, our study demonstrated that crystalline modification of nanoscale TiO_2_ can determine the pattern of cell response to the treatment. This is the first direct evidence in favor of the early proposed statement that nanosizes and nanodistances could generate principally new types of cell responses to inorganic substances. 

## 4. Summary

We synthesized nanoscale TiO_2_ sols with different textural and structural-dispersion characteristics of nanoparticles by varying the nature of TiO_2_ precursor and conditions of hydrolysis and made their comparative analysis using a set of physicochemical methods. pH of the medium and temperature were shown to be the key factors determining the formation of TiO_2_ structure during hydrolysis of TiO_2_ precursor. The selected methods of synthesis make it possible to prepare the amorphous, anatase, brookite, and rutile modifications of TiO_2_ nanoparticles with prevailing diameter 3–5 nm. All modifications of TiO_2_ nanoparticles have similar dispersion and morphological characteristics (apart from rutile), which do not change in a cell culture medium. A comparison of the particle size measured by XRD, SAXS, DLS, AFM, and HR-TEM showed good agreement between data obtained by these methods. The data are complementary and provide comprehensive and reliable information on the particle size and morphological features. 

Electron microscopy of ultrathin sections of MDCK cells incubated with different forms of TiO_2_ nanoparticles revealed the direct contact of single amorphous, anatase, and brookite nanoparticles with the cell surface and subsequent different cell responses to the treatment. The TiO_2_ modification determines the pattern of cell reaction to the nanoparticles. Direct contact of amorphous, anatase, and brookite single nanoparticles with the cell plasma membrane could alter its function by a simple mechanical binding to membrane macromolecules. However, the influence of TiO_2_ nanoparticles on cell functions is related most likely to the chemical reactions between the particles and cell membrane macromolecules. We suppose that direct contact of the nanoparticles with cell plasma membrane is the primary and critical step of their interaction, which determines a subsequent response of the cell. 

## Figures and Tables

**Table 1:** Synthesis conditions of titanium dioxide samples and their properties.

Samples	Hydrolysis conditions		Textural and structural-dispersion properties of TiO_2_				
	*T*, °C	pH	*S*_BET_, m^2^·g^−1^	*V*_pore_, cm^3^·g^−1^	Phase composition	Crystallite size, nm	*D*, nm (SAXS)
1.1	4	3-4	330	0.2	Anatase	5.4	5.8
1.2	4	6-7	360	0.6	X-ray amorphous	—	7.0
1.3	4	9-10	420 (400)^a^	0.5	X-ray amorphous	—	5.0
1.4	20	3-4	350 (330)^a^	0.3	Anatase	5.4	5.2
1.5	20	6-7	400	0.5	X-ray amorphous	—	5.0
1.6	20	9-10	495	0.4	X-ray amorphous	—	5.3
1.7	70	3-4	380	0.6	Anatase	4.3	6.1
1.8	70	6-7	510	0.5	X-ray amorphous	—	5.6
1.9	70	9-10	525	0.4	X-ray amorphous	—	5.4
2.1	4	0.2	70 (850)^a^	0.05	Brookite (the main phase)	2.3	2.2
2.2	70/1 h	0.2	100	0.1	Brookite + anatase	2.8	3.1
2.3	70/5 h	0.2	175 (200)^a^	0.17	Rutile	7.0	7.0
3.1	20	1.2	2	0.01	Anatase	3.0	3.0
3.2	50	1.2	60	0.04	Anatase	4.2	4.5
3.3	70	1.2	185 (300)^a^	0.10	Anatase	4.7	5.2
3.4	95	1.2	230	0.13	Anatase	5.4	5.8
3.5	60	1.2	1.8 (530)^a^	0.01	Anatase	3.3	2.8

^a^S_SAXS_.

**Table 2:** Hydrodynamic diameters of TiO_2_ particles in freshly prepared and neutralized sols.

Samples	Particle size (nm) in fresh sols		Particle size (nm) in neutralized sols
	Before US	After US	After US
Amorphous (1.5)	12000	202	200
Brookite (2.1)	20	2.2	50
Rutile (2.2)	1200	1200	1200
Anatase (3.2)	10	12	90
Anatase (3.3)	7	13	40
Anatase (3.5)	3	9	50
